# Skin melanin is associated with body temperature regulation in humans and mice

**DOI:** 10.1371/journal.pone.0334735

**Published:** 2025-11-07

**Authors:** Kale S. Bongers, Santiago Tovar, Zanthia Wiley, Michele Sumler, Nina G. Jablonski, Adewole S. Adamson, Sivasubramanium V. Bhavani

**Affiliations:** 1 Division of Pulmonary, Critical Care, and Occupational Medicine, Department of Internal Medicine, Carver College of Medicine, University of Iowa, Iowa City, Iowa, United States of America; 2 Division of Pulmonary and Critical Care Medicine, Department of Internal Medicine, University of Michigan, Ann Arbor, Michigan, United States of America; 3 Department of Medicine, Emory University, Atlanta, GeorgiaUnited States of America; 4 Department of Anesthesiology, Emory University, Atlanta, GeorgiaUnited States of America; 5 Department of Anthropology, Pennsylvania State University, University Park, Pennsylvania, United States of America; 6 Department of Internal Medicine, Dell Medical School, Austin, Texas, United States of America; 7 Emory Critical Care Center, Emory University, Atlanta, GeorgiaUnited States of America; Wenzhou Medical University, CHINA

## Abstract

Body temperature, a universally measured clinical indicator of physiological equilibrium, guides critical treatment decisions. Multiple studies have observed significant body temperature differences among racial subgroups, with Black patients consistently having higher temperatures than White patients. However, race is a social construct and not a biological category; thus, race alone cannot explain this temperature variability. We hypothesized that skin melanin, which often varies across racial categories, could explain body temperature differences. Here, using a prospectively enrolled human cohort study and a parallel mouse model, we demonstrate that skin melanin is associated with body temperature in humans and mice. In humans, colorimeter-measured melanin index was positively correlated with temperature. Likewise, we found that pigmented mice had higher temperatures than albino mice. Our results reveal that melanin could explain the consistent differences in body temperature observed across socially defined racial groups and suggest a potential role for melanin in thermoregulation.

## Introduction

Thermoregulation is a fundamental physiological process in health and disease. Over the past few decades, studies have noted significant differences in body temperature across racial subgroups in the U.S., with the consistent finding of higher oral temperatures in Black patients compared to White patients [1–5]. Race is a social construct and not a biological category [6]. Thus, racial designations do not explain these body temperature differences. However, skin melanin expression varies across populations and has been historically linked with socially defined racial categories [7,8]. Rather than the social construct of race, the biological variable of melanin may explain the observed differences in temperature.

To test whether melanin is associated with body temperature, we performed two distinct investigations: 1) in a prospective human clinical study, we evaluated the association between quantified skin melanin and oral temperature; 2) in mouse model studies, we compared the differences in temperature between albino mice (which produce no melanin) and pigmented mice (which produce melanin).

## Methods

### Human clinical study

Adult patients admitted to three medical ICUs at two different hospitals in Atlanta, GA, USA were eligible for enrollment during the period of 01/01/2024–31/12/2024. Demographic information including self-reported race as documented in the Electronic Health Record was noted. Geographic ancestry information was not available. Oral temperature was measured with a Welch Allyn thermometer. Skin tone was measured on the forehead using the Delfin colorimeter to obtain two metrics: 1) melanin index, and 2) Individual Typology Angle (ITA). Melanin index is a unitless measure of the reflectance ratio of the skin, with higher values for darker skin tone and ITA is a standardized metric calculated based on the L*a*b color space, with lower values for darker skin tone [9]. All measurements were taken between 11am to 5 pm. Covariates of interest that could affect body temperature were recorded including age, sex, requirement of vasopressors, mechanical ventilation, and continuous renal replacement therapy, and timing of temperature measurement. The agreement between melanin index and ITA was assessed using the intraclass correlation coefficient (ICC). The association between oral temperature and each of the two skin tone measures was evaluated using Pearson correlation coefficients. To further examine the relationship between oral temperature and melanin index, multivariable linear regression was performed, adjusting for age, sex, vasopressor use, mechanical ventilation, continuous renal replacement therapy, and timing of temperature measurement. The study was approved by the Institutional Review Board at Emory University (Study #5545), with a waiver of documentation of informed consent (i.e., signature). Verbal consent was obtained from participating subjects or their legally authorized representatives using the IRB-approved consent script, and the process was documented by the clinical research coordinator in the study records at the time of enrollment. Informed consent was not waived; only the requirement for a subject’s signature was waived, as approved by the IRB.

### Mouse models

Seven-week-old female C57BL/6 and B6(Cg)-*tyr*^*c-2J*^ mice were obtained from Jackson Laboratories (Bar Harbor, ME). As described previously [10], mice were housed at 21°C in colony cages with a 12:12-hour light-dark cycle with *ad libitum* access to water and standard chow (Envigo Teklad, Indianapolis, IN). Rectal temperatures were measured at the same time of day with a Fisherbrand™ Traceable™ thermometer with RET-3 mouse rectal probe (ThermoFisher Scientific, Waltham, MA). For non-mixing studies, mice were kept isolated by strain and left to equilibrate for 7 days prior to temperature measurement. For subsequent mixing studies, mice were mixed so that each cage contained mice from each strain for 14 days, at which time body temperatures were again measured. Importantly, prior studies demonstrate microbiome mixing by 7 days of co-housing [11,12]. All animal studies were approved by the Institutional Animal Care and Use Committees of the Universities of Michigan (PRO00009673) and Iowa (#4052592). Mice were euthanized via carbon dioxide asphyxiation followed by cervical dislocation for confirmation, as per AVMA guidelines. Additional anesthesia and analgesia methods were not used for rectal temperature measurement or euthanasia.

## Results

### Human clinical study

**Fig 1:**
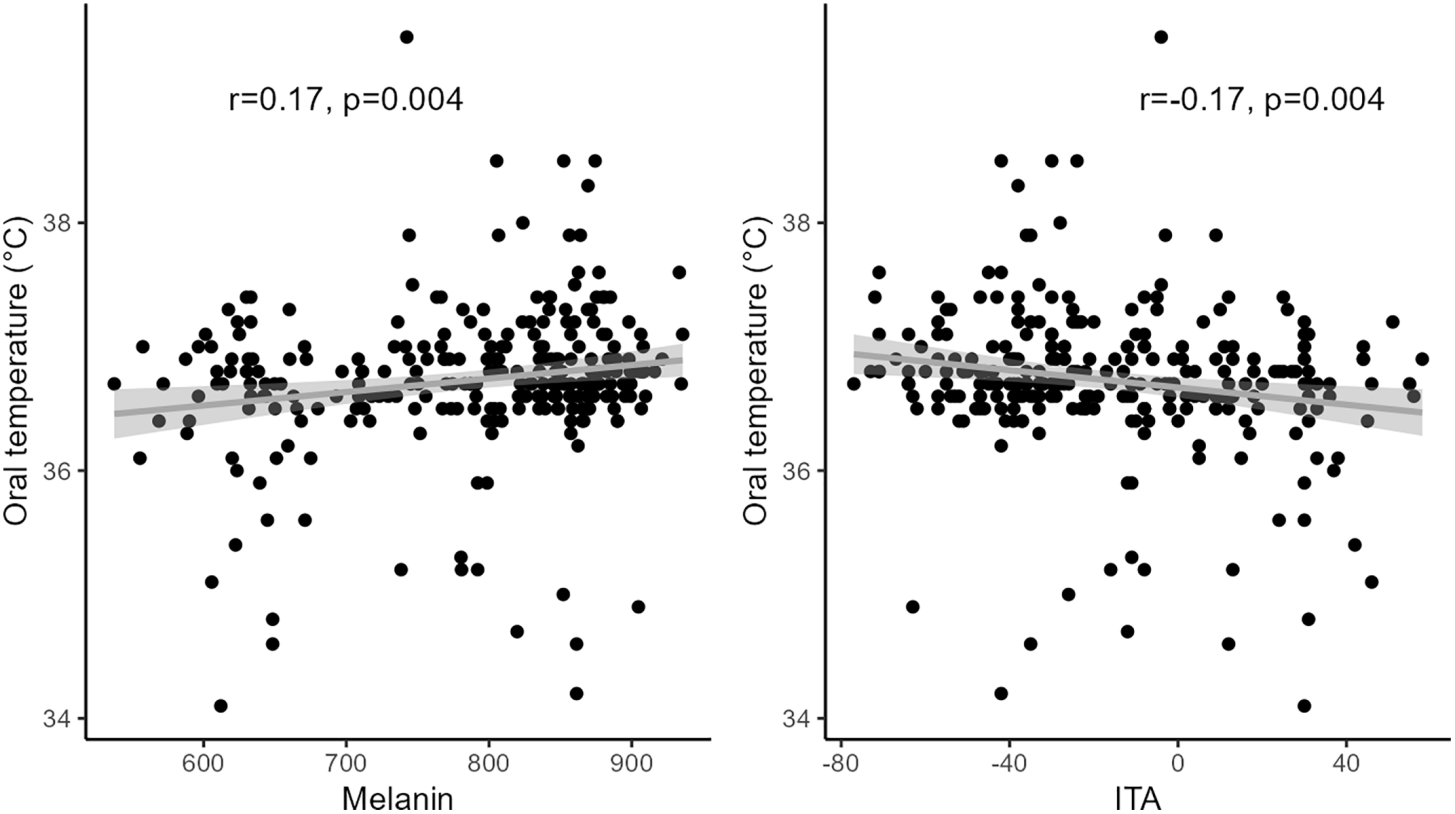
Correlation between melanin and oral temperature. Melanin index and Individual Typology Angle (ITA) are plotted against oral temperature in 275 hospitalized patients, and the regression fit with 95% confidence interval is shown. Melanin and ITA were both significantly correlated with oral temperature.

In the prospective study of 275 hospitalized patients, the median age was 62 years (IQR 47−73 years), 54% males, and 74% Black, 19% White, and 7% other race. Melanin and ITA were significantly correlated (r = −0.95, p < 0.001). Both melanin and ITA were significantly correlated with oral temperature (r=+0.17, p = 0.004 and r = −0.17, p = 0.004, respectively) (Fig 1). When adjusting for age, sex, presence of vasopressors, mechanical ventilation, or renal replacement therapy, and timing of measurement, melanin index and ITA remained significantly associated with oral temperature (p = 0.03 and p = 0.04, respectively). When excluding patients with a fever (temperature>38.3°C), melanin and ITA remained significant associated with oral temperature (p = 0.02 and p = 0.03, respectively). When the models were adjusted for race, neither melanin nor race were significantly associated with body temperature. To disentangle the role of melanin from the construct of race in association with temperature, we performed the following mouse model.

### Mouse model

**Fig 2:**
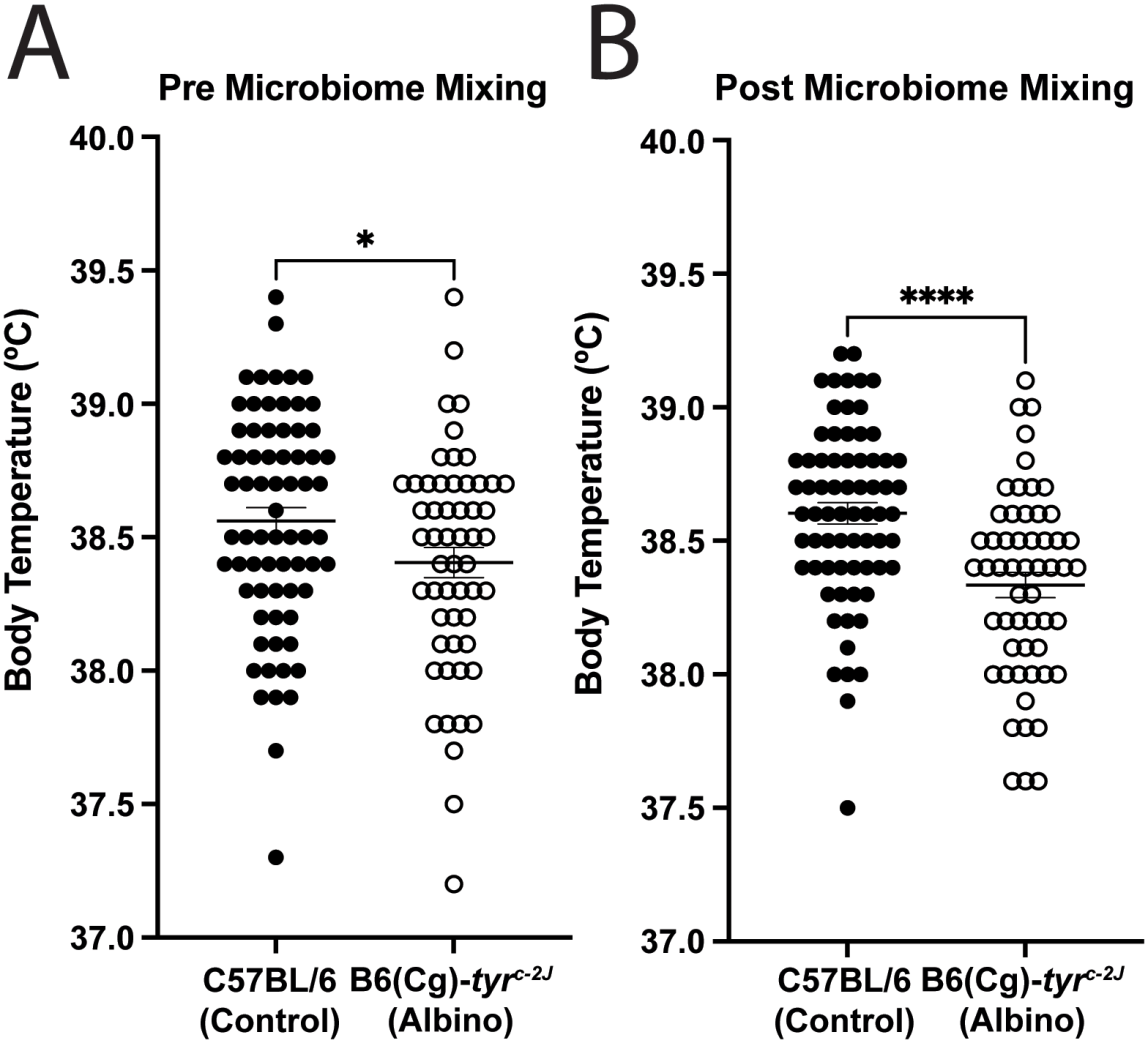
Skin pigmentation mediates body temperature in mice. **(A-C)** Female C57BL/6 and B6(Cg)-*tyr*^*c-2J*^ mice were obtained from Jackson Laboratories, and temperature was assessed with a rectal thermometer. Error bars denote SEM. **(A)** B6(Cg)-*tyr*^*c-2J*^ have lower basal body temperature compared to C57BL/6 mice prior to microbiome mixing. n = 55-70 mice per treatment. *P* < 0.04. **(B)** After microbiome mixing, B6(Cg)-*tyr*^*c-2J*^ continued to have lower basal body temperature compared to C57BL/6 mice. n = 55-70 mice per treatment. *P* < 0.0001.

To evaluate whether these associations were due to pigmentation or merely an epiphenomenon, we used a mouse model to minimize biological and environmental variability which might confound the human study. Utilizing C57BL/6 mice (which are pigmented) and B6(Cg)-*tyr*^*c-2J*^ mice (which are on the C57BL/6 genetic background but are homozygous for a tyrosinase mutation rendering them albino [13]), we compared baseline body temperatures. Similar to the finding of higher body temperature in patients with higher melanin content, we found the pigmented C57BL/6 mice had a significantly higher body temperature than the otherwise genetically-matched albino B6(Cg)-*tyr*^*c-2J*^ strain (38.6 ± 0.41°C versus 38.4 ± 0.42°C, *P* < 0.04) (**Fig 2A**). Since the gut microbiome can influence body temperature in mice [10], and gut microbiota can vary by mouse shipment [14,15], as an additional control we minimized microbiome effects via a two-week cage mixing study (a duration previously shown to equilibrate gut microbiota [11,12]), and again found that the pigmented C57BL/6 mice had a significantly higher body temperature than albino B6(Cg)-*tyr*^*c-2J*^ mice (38.6 ± 0.33°C versus 38.3 ± 0.35°C, *P* < 0.0001) (**Fig 2B**).

## Discussion

This paper presents novel evidence of a relationship between melanin concentration and temperature through both human and mouse studies, in which we found that: 1) higher levels of melanin were associated with higher oral temperatures in humans; and 2) pigmented mice had higher rectal temperatures than albino mice on the same genetic background. Over several decades, studies have consistently noted higher oral temperatures in Black patients compared to White patients in the U.S [1–5]. Since race is a social construct and not a biological category [6], race by itself is unlikely to explain this difference in temperature measurement. However, in human clinical studies, race and skin tone are often closely intertwined, and it is difficult to disentangle the role of melanin in thermoregulation [7]. The mouse model provides evidence that melanin may play a role in thermoregulation.

While the temperature differences we found were relatively small in absolute terms, given the relatively narrow homeostatic range of body temperature (approximately 2°C), this is a potentially clinically relevant change. Additionally, given the typical “all or none” clinical response to fever, where a certain temperature threshold (38.3°C in ICU patients [16]) often triggers changes to treatment (such as empiric antibiotics or antipyretics), slight temperature differences may lead to outsized clinical effects at a healthcare-system level.

These findings have important implications for our understanding of body temperature regulation. Thermoregulation is complex and involves the neurological, cardiovascular, musculoskeletal, endocrine, and immune systems [17,18]. However, the role of epidermal melanin in thermoregulation remains poorly understood. Melanin is produced in melanocytes from the amino acid tyrosine through a process beginning with an oxidation step by the enzyme tyrosinase [19]. Melanin is then transported to keratinocytes in the skin, where it serves to absorb ultraviolet light and near-infrared radiation to protect the underlying tissue [19–21]. Through this role, melanin plays a direct part in heat transfer in endothermic species [22–24], and thus could be involved in thermoregulation. Other potential mechanisms are possible. For example, melanin is associated with a reduced capacity for Vitamin D synthesis, particularly under conditions of limited UVB exposure. Additionally, melanin helps protect bioactive folates from photodegradation, which may have downstream effects on physiological processes [25]. These molecules could potentially mediate thermoregulatory pathways. Furthermore, melanin’s precursor tyrosine is also a precursor to catecholamine synthesis [26,27]. Small human studies have shown that tyrosine supplementation reduces temperature drop during cold tolerance testing through increased cutaneous vasoconstriction [26,27], which could suggest a tradeoff between melanin and catecholamine production regulating body temperature. Further studies are needed to investigate these mechanistic hypotheses. In particular, evaluating potential body temperature changes in mice with genetically-inducible melanin would be useful.

Beyond melanin itself, other melanin-regulating pathways may also play a role in thermoregulation. First, melanocyte-stimulating hormone (MSH) stimulates melanogenesis in human melanocytes [28–30] and MSH administration can prevent fever in septic rabbits through effects on prostaglandin synthesis [31] and induce hypothermia in rats [32]; MSH receptor agonists have biphasic effects on body temperature [33]. Second, melanin-concentrating hormone (MCH) also plays an important role in skin pigmentation in some species of fish [34], while in mammals, antibodies blocking the MCH receptor appear to play a role in vitiligo pathogenesis [35]. Knockout of the genes encoding MCH [36] and the MCH receptor [37] increases basal body temperature, while exogenous MCH infusion reduces body temperature [38]. Third, the gene *KIT Ligand (KITLG)* is also known to be both involved in melanogenesis and a regulator of thermogenesis in brown adipose tissue [39]. Additional studies are needed to explore relationships between melanogenesis and thermogenesis, which may involve several of these interrelated pathways.

Our study has several limitations. First, oral temperatures may not reflect core body temperatures, and different sites of measurement may have different associations with melanin. Second, our human melanin quantification study took place in ICUs, and critically ill patients may have different thermoregulatory responses from the general population. Third, geographic ancestry of the patients was not ascertained, and while race is a social construct, ancestry is likely more closely linked to differences in melanin. Fourth, there may be unmeasured confounders associated with both race and melanin in our human study which could explain temperature differences. Fifth, confounders such as ambient temperature were not measured and adjusted for. Finally, our mouse models used rectal rather than oral temperature measurements, as oral temperature measurement is not a validated method in mice [40]. This difference in measurement sites (oral temperatures in humans, but rectal temperatures in mice) may affect comparability of the results. Future studies using human rectal temperature measurements and alternate methods of murine temperature measurement, particularly long-term surgically-implanted monitors [40], would be informative.

In conclusion, higher melanin levels are associated with higher oral temperature in humans and higher rectal temperature in mice, suggesting a potential role of melanin in thermoregulation.

## Supporting information

S1 Table De-identified patient-level information on melanin, ITA, and oral temperature. (CSV)
